# Effect of different surface treatments on retention and fracture strength of zirconia crowns over Ti-base zirconia abutments

**DOI:** 10.1016/j.heliyon.2024.e40164

**Published:** 2024-11-05

**Authors:** Mehran Falahchai, Hamid Neshandar Asli, Mehdi Daneshvar, Amirreza Hendi

**Affiliations:** Dental Sciences Research Center, Department of Prosthodontics, School of Dentistry, Guilan University of Medical Sciences, Rasht, Iran

**Keywords:** Zirconia abutments, Zirconia crown, Fracture strength, Retention

## Abstract

**Background:**

Limited evidence suggested different outcomes of surface treatment methods on zirconia abutments and crowns. Therefore, we investigated the effect of grooving, hot etching, and airborne particle abrasion (APA) methods on zirconia crowns over Ti-base zirconia abutments retention and fracture strength.

**Materials and methods:**

In this *in vitro* study, 110 zirconia crowns and abutments were divided into five groups, including APA for crown and grooved zirconia abutment (APACr-GrAb), APA for crown and hot etching zirconia abutment (APACr-HtAb), grooved modified zirconia crown and APA for zirconia abutments (GrCr-APAAb), hot etching modified zirconia crown and APA for zirconia abutments (HtCr-APAAb), and APA for both crown and zirconia abutments (control group). The retention and fracture strength were measured. Data were analyzed by ANOVA, Tukey, Kruskal-Wallis, and Mann-Whitney with Bonferroni correction tests (α = 0.05).

**Results:**

Hot etching and grooving on the crown or abutment significantly resulted in higher retention (P < 0.001). The fracture strength significantly differed among the five groups (P = 0.041), with the highest fracture strength observed in the GrCr-APAAb group and the lowest for the APACr-GrAb group; however, no significant differences were found in pairwise comparisons between groups (P > 0.05). By considering both fracture strength and retention, grooving, hot etching, and APA were confirmed for better crown and abutment function, respectively.

**Conclusion:**

Grooving surface treatment was the best method due to its high fracture strength and retention, followed by hot etching due to its high retention compared to APA, which can be considered a suitable method for cementing zirconia crown on zirconia abutments.

## Highlights

• Hot etching and grooving significantly improved retention.
• Grooving and APA treatments result in the highest fracture strength, suggesting they strengthen the structural integrity of zirconia crowns on zirconia abutments.
• Grooving surface treatment was the best method due to its high fracture strength and retention.

## 1 Introduction

The quest for optimal dental restorations has led to significant advancements in materials and techniques, among which zirconia (zirconium dioxide, ZrO_2_) stands out due to its optimal aesthetic properties, high fracture strength, and superior biocompatibility [1]. Implant-supported zirconia restorations have become increasingly popular, offering a robust and aesthetically pleasing solution for patients requiring dental implants [2,3]. However, the success of these restorations heavily relies on their retention and fracture strength, which can be influenced by various surface treatment methods applied to the zirconia [4,5]. Bond strength to different types of cement is considered a limiting factor for using zirconia crowns [6,7], and various chemomechanical surface preparation methods were demonstrated to improve the cement/zirconia bond mechanism [8].

Zirconia crowns, often used in dental restorations, benefit from various modifications and surface treatments designed to enhance their performance [9]. Zirconia abutments on Titanium (Ti) Ti-bases combine the aesthetic benefits of zirconia with the mechanical strength of titanium, providing an optimal interface for dental implants [10,11].

Different surface treatments for zirconia crowns and abutments include grooving, airborne particle abrasion (APA), chemical agents, hot etching, etc. [12]. APA, also known as sandblasting, is a widely used method that involves propelling aluminum oxide particles against the zirconia surface under high pressure. This process creates micro-retentions and increases the surface area available for bonding [13,14]. On the other hand, chemical agents and hot etching techniques represent alternative approaches to zirconia surface modification. Chemical treatments often involve the application of hydrofluoric acid, phosphoric acid, or other etching solutions to selectively remove surface atoms and create a more reactive surface [[15], [16], [17]]. Hot etching, a recent development that utilizes a molten salt bath at elevated temperatures to aggressively etch the zirconia surface, has shown promise in creating a highly retentive surface topography without compromising the material's structural integrity [18,19]. Evidence shows that grooving involving the creation of micro retentive ridges or grooves on the zirconia abutment, significantly improves bond strength by increasing surface area and providing mechanical retention points [20,21].

The choice of method depends on the specific clinical scenario, desired outcomes, and available resources. Although numerous studies investigated the effects of various surface treatment methods on zirconia, there has been no focused research specifically on zirconia crowns over Ti-base zirconia abutments based on author's knowledge [8,12,16]. Furthermore, existing information on techniques like hot etching remains scarce and often contradictory [18,19]. This gap in the literature highlights the need for our study, Therefore, the current study compared the effectiveness of grooving, hot etching, and APA methods on zirconia crowns over Ti-base zirconia abutments retention and fracture strength. The null hypothesis of the study is that different surface treatments do not influence retention and fracture strength of zirconia crowns over Ti-base zirconia abutments.

## 2 Materials and methods

### 2.1 Study design

This *in vitro* study was confirmed by the ethical committee of the Guilan University of Medical Sciences [IR.GUMS.REC.1399.660]. Considering the fracture strength variable for the studied groups, the statistical power was 0.85, the error level was 0.05, and the standard deviation (SD) was 9.60. A minimum sample size of 10 was obtained for each group [22]. Sample size using calculated from the ANOVA section of PASS version 11 software.

### 2.2 Surface treatment

**Fig. 1:**
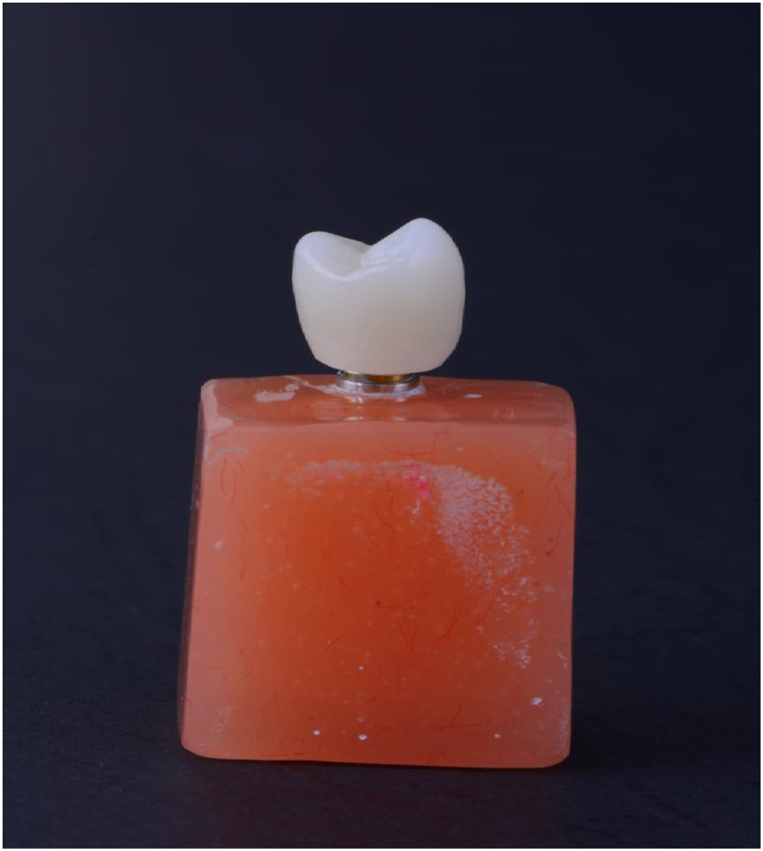
The zirconia specimen for the fracture strength test.

**Fig. 2:**
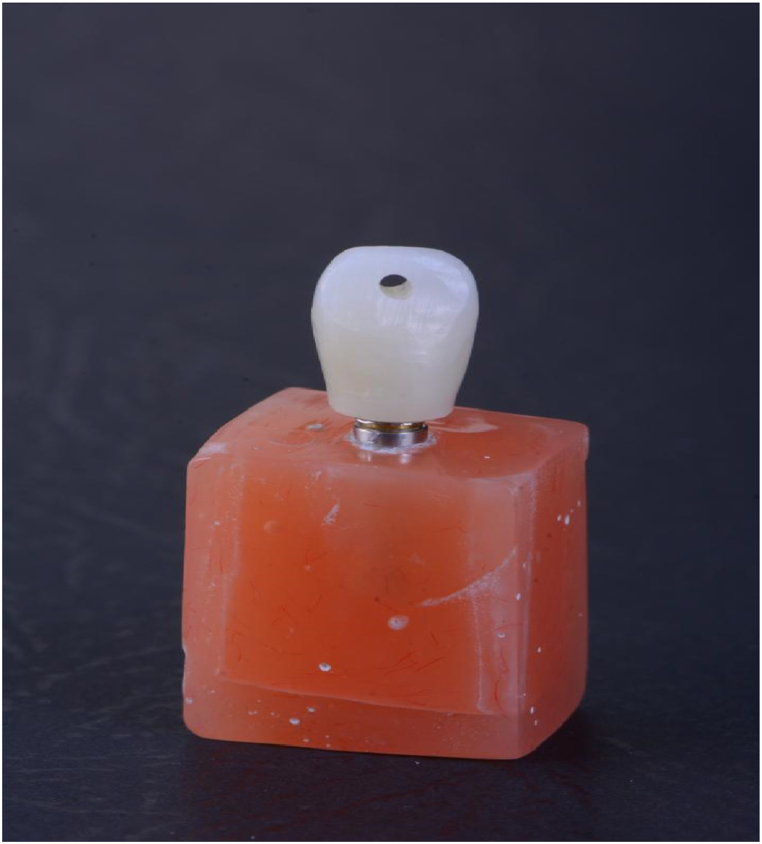
The zirconia specimen for the retention test.

To evaluate the effectiveness of different surface preparation methods on the retention and fracture strength of zirconia crowns cemented onto Ti-base zirconia abutments, 110 SIC implant analog fixtures (SIC invent AG, Switzerland), along with pre-sintered zirconia blocks (Ceramill Zolid, Amann Girbach, Germany) for milling 110 abutments and 110 crowns were used each with a thickness of 2 mm. A cylindrical Ti-base zirconia abutment with a height of 4 mm, an access hole, and a 30-μm cement space on the inner surface was designed using software (Ceramill Mind, Amann Girbach, Germany). Then, using the same software, a full-contour crown for the first maxillary premolar was designed along with a 30-μm cement space (Fig. 1). In specimens for retention test, for a better connection to the Universal Testing Machine (SANTAM, model DBBP50, Korea) on the occlusal surface, a 2 mm zirconia loop was designed and sent to the milling machine (Ceramill Motion 2 (5x), Amann Girrbach, Germany) (Fig. 2). According to the manufacturer's instructions, all abutments and crowns were sintered at 1480 °C for 8 h in a sintering furnace (Ceramill Therm, Amann Girbach, Germany). The specimens were placed in an ultrasonic device containing distilled water at 37 °C for 5 min and then divided into five equal groups.

**Fig. 3:**
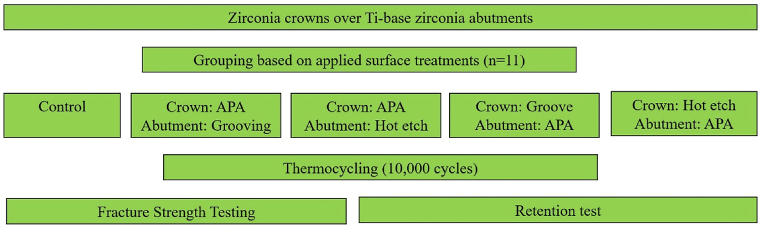
Schematic diagram of experimental procedures (APA: airborne-particle abrasion).

The first group consisted of APA crown and grooved zirconia abutment (APACr-GrAb). The second group comprised APACr and hot etching zirconia abutment (APACr-HtAb). The third group consisted of grooved modified zirconia crowns and APA zirconia abutments (GrCr-APAAb). The fourth group consisted of hot-etched modified zirconia crowns and APA zirconia abutments (HtCr-APAAb), and the control group included APA crowns with APA zirconia abutments [23] (Fig. 3). The APA treatment involved surface modification using air abrasion. An intraoral device (Microsandblaster; Dento‐Prop Ronvig, Daugård, Denmark) propelled 25-μm aluminum oxide particles onto the sample. The process was conducted with the nozzle held perpendicular to the surface, maintaining a 10-mm gap. The abrasive stream was applied for 20 s at 0.25 MPa [12]. To create a groove, diamond process bur with a low-speed handpiece without the presence of water (6801, Komet Besighgehim, Germany), grooves with a width and depth of 0.5 mm were created in the mesial and distal abutment up to 0.5 mm of the finish line before sintering on the zirconia. For hot etching, the specimens were immersed in a hot acidic solution containing 800 ml of methanol, 200 ml of HCl, and 2 gr of FeCl_3_, heated to 100 °C for 30 min [24].

The cubic acrylic blocks (Cold-cure Acrylic for repair, Marlic Dental, Iran) measuring 11x11 × 11 mm were made by molds with base plate wax. Analog implants were mounted using a survivor inside the block with an angle of 90° to the horizontal axis and in the condition that the contact of the abutment and analog fixture was 1 mm above the acrylic surface. Then, the Ti-base abutments were torqued to 30 N-cm. After outer surface modifications, zirconia abutments were cemented on Ti-base abutments using Duo-Link resin cement (Bisco Inc., Schaumburg, USA). The surface-modified crowns were cemented with Duo-Link resin cement (Bisco Inc., Schaumburg, USA) according to the manufacturer's instructions. Hand pressure was applied for 20 s on all four sides using a Bluephase G2 light curing unit (Ivoclar Vivadent, 1200 mW/cm^2^) [25]. The samples were subjected to repeated temperature fluctuations between 5 and 55 °C. This process was carried out 10,000 times, with each cycle consisting of a 30-s exposure period at each temperature extreme and a 20-s transition interval [12]. After thermocycling, the specimens were stored in distilled water at 37 °C with 100 % humidity for 24 h.

### 2.3 The retention and fracture strength tests

The retention and fracture strength was assessed using a Universal Testing Machine (SANTAM, model DBBP50, Korea). The implant analogs were secured inside a chamber at the bottom of the machine for retention measurement. A high-resistance metal wire with a diameter of 2 mm was looped through the hole in the zirconia crown. This wire loop was connected to the front of the machine, and the crowns were separated from the abutments at a speed of 1 mm/min under tensile force along the vertical axis of the abutment. The maximum force before the complete debonding of the cemented crowns from the abutments was measured and recorded as the retention force. To assess the fracture strength, the compressive force was applied vertically at a 0° angle to the occlusal surface of the crown at a speed of 1 mm/min. The force intensity was increased until the crown was fractured, and the fracture strength force was recorded.

### 2.4 Statistical analysis

To analyze the data, the presuppositions of the ANOVA parametric test were first examined. The normality of the investigated groups was examined using the Shapiro-Wilk test, and the variance homogeneity of the groups was examined using Levene's test. An analysis of variance test was used to determine the strength variable according to the assumptions. The Kruskal-Wallis test was used for the retention variable due to the non-normality of one of the groups. Tukey's test was used for ANOVA pairwise comparisons for fracture variables, and the Mann-Whitney test with Bonferroni correction was used for Kruskal-Wallis's paired comparisons due to the presence of five groups. All data was analyzed using SPSS version 28 (IBM SPSS Statistics, IBM Corp., NY, USA), and a significant level was set at 0.05. Power calculations were performed with G-Power version 3.1.9.2 software as 1.00.

## 3 Results

Our findings based on the Kruskal-Wallis Test showed a significant difference in mean retention rate between the five groups (P < 0.001). The highest mean retention was observed in the APACr-HtAb group (220.25 ± 20.69 N), while the lowest mean (171 ± 17.79 N) was in the control group (Table 1). According to our findings, grooving and hot etching enhanced the retention regardless of the substrate they applied. Further analysis using the Mann-Whitney test with Bonferroni correction showed that the control group had significantly lower retention than the other groups (P < 0.05). This confirms that APA alone is less effective than when combined with other surface treatments like hot etching or grooving (Table 2).

**Table 1:** The results of determining and comparing the average retention rate and fracture strength of zirconia crowns based on cemented implants on Ti-base zirconia abutments according to surface preparation methods.

Group	Retention						Fracture strength				
	Mean ± SD (N)	Mean Rank	Statistics	95 % Confidence Interval		P value^a^	Mean ± SD (MPa)	Statistics	95 % Confidence Interval		P value^b^
				Lower bound	Upper bound				Lower bound	Upper bound	
**APACr-GrAb**	217.50 ± 21.93	34.63	24.07	203.56	231.43	<0.001	1182.30 ± 106.65	2.7	1106.00	1258.59	0.041
**APACr-HtAb**	220.25 ± 20.69	38.43		207.10	233.39		1098.30 ± 73.01		1046.07	1150.52	
**GrCr-APAAb**	217.17 ± 23.63	35.63		202.15	232.18		1203 ± 112.20		1122.73	1283.26	
**HtCr-APAAb**	215.58 ± 18.53	35.29		203.81	227.35		1100.40 ± 76.83		1045.43	1155.36	
**Control group**	171 ± 17.79	8.54		159.69	182.30		1111.50 ± 101.01		1039.24	1183.75	

Groups included airborne particle abraded (APA) crown and grooved zirconia abutment (APACr-GrAb), APACr and hot etching zirconia abutment (APACr-HtAb), grooved modified zirconia crown and APA zirconia abutments (GrCr-APAAb), hot etched modified zirconia crown and APA zirconia abutments (HtCr-APAAb), and APA crown with APA zirconia abutments (control group).

a Kruskal-Wallis Test.

b ANOVA.

**Table 2:** Pair-by-pair comparisons of average retention rate and fracture strength of zirconia crown based on cemented implants on Ti-base zirconia abutments according to surface preparation methods.

Group		APACr-GrAb	APACr-GrAb	APACr-GrAb	APACr-GrAb
a**Retention**	**APACr-GrAb**	–	–	–	
	**APACr-HtAb**	0.999	–	–	–
	**GrCr-APAAb**	0.999	0.999	–	–
	**HtCr-APAAb**	0.999	0.999	0.999	–
	**Control group**	<0.003	<0.001	0.001	0.002
b**Fracture strength**	**APACr-GrAb**	–	–	–	–
	**APACr-HtAb**	0.296	–	–	–
	**GrCr-APAAb**	0.988	0.119	–	–
	**HtCr-APAAb**	0.321	0.999	0.132	–
	**Control group**	0.467	0.998	0.219	0.999

Groups included airborne particle abraded (APA) crown and grooved zirconia abutment (APACr-GrAb), APACr and hot etching zirconia abutment (APACr-HtAb), grooved modified zirconia crown and APA zirconia abutments (GrCr-APAAb), hot etched modified zirconia crown and APA zirconia abutments (HtCr-APAAb), and APA crown with APA zirconia abutments (control group).

a Mann-Whitney test with Bonferroni correction.

b Tukey HSD.

The ANOVA test indicated a significant difference in fracture strength across the groups (P = 0.041). The GrCr-APAAb group recorded the highest mean fracture strength at 1203 ± 112.20 MPa, significantly higher than the APACr-HtAb group (1098.30 ± 73.01 MPa). Although some groups' differences in fracture strength were not statistically significant, the data suggests that grooving, particularly when paired with APA, provides an optimal balance between retention and fracture strength (Table 1). Results illustrated that hot etching on zirconia crowns, and abutments led to reduced fracture strength, and the highest fracture strength was observed in grooving and APA treatment, which has been observed in GrCr-APAAb and APACr-GrAb groups, respectively.

## 4 Discussion

Zirconia crowns and abutments are widely used in dental restorations due to their superior aesthetic qualities and exceptional mechanical properties. However, achieving optimal retention and fracture strength remains a critical challenge [4,26]. Various surface modification techniques have enhanced zirconia restorations' bonding and mechanical performance. These surface treatments alter the zirconia's surface characteristics, potentially improving its interaction with luting agents and overall durability [27,28]. The current study investigated the retention and fracture strength differences of zirconia crowns and abutments after applying surface treatment by APA, grooving, and hot etching. The findings demonstrated a significant difference in the retention and fracture strength of zirconia crowns and abutments, in which applying grooving and hot etching enhanced the fracture strength and retention, illustrating the importance of utilizing preparing methods. Thus, the null hypothesis of this study was rejected.

The current study showed that grooved abutments resulted in higher retention and fracture strength than APA abutments. The retention of dental restorations is significantly influenced by the surface characteristics of the abutment and the type of luting agents utilized [29]. Recent studies have confirmed the importance of surface treatments in enhancing the retention and fracture strength of zirconia crowns. For instance, research has shown that mechanical surface treatments like airborne-particle abrasion (APA) with alumina and tribochemical silica coatings significantly improve the long-term retention of zirconia crowns, especially after aging processes such as thermal cycling [30,31]. This aligns with the findings in your study, where APA combined with grooving or hot etching resulted in superior retention and fracture strength.

Moreover, newer methods, like selective infiltration etching (SIE) and novel glass-ceramic spray deposition, have demonstrated promising results in increasing the durability and bond strength of zirconia to resin cement. However, these methods require extensive testing and are not yet widely adopted in clinical practice [32]. The observation that hot etching was more effective in improving retention but not fracture strength compared to APA is also supported by recent studies. These studies suggest that while hot etching creates a more reactive surface for bonding, it may introduce microstructural weaknesses that could reduce overall fracture strength [32]. Sahu et al. reported that surface treatment of the abutment through grooves or APA can improve retention, particularly in short clinical crowns. These modifications created a more textured surface, which increased the mechanical interlocking between the abutment and the restorative material. Consequently, this led to a more secure and durable bond, ensuring the stability and longevity of the restoration even under the challenging conditions presented by short clinical crowns [33].

The differences in abutment and coping materials, the orientation of the grooves, and the type and method of cementation all contribute to variations in outcomes. This study attributed the increased retention observed in grooved abutments to mechanical interlocking. Similarly, the increased retention with the APA method was due to the enhanced surface area, which facilitates micromechanical bonding. A study by Kevam et al. demonstrated that the adhesion strength of resin cement to zirconia varies significantly with different surface treatments. Melt-etching with potassium hydrogen difluoride (KHF2) produced the highest adhesive strength, followed by grinding with a carbide bur, and APA resulted in the lowest adhesive strength. Therefore, for enhanced adhesion of resin cement to zirconia, melt-etching with KHF2 is the most effective surface treatment compared to traditional methods like APA and grinding [8].

Due to our findings, hot etching was more effective for increasing retention but not for fracture strength than the APA method. Lv et al. showed that zirconia ceramics prepared with hot etching had greater bond strength of zirconia to resin than those prepared with APA, regardless of the type of cement used. They found that the bond strength increased with longer etching times, with 60 min of hot etching resulting in the highest bond strength, followed by APA, 30 min of hot etching, and 10 min of hot etching [19]. Tanis et al. reported that the selective infiltration etching (SIE) technique significantly enhanced the shear bond strength of resin cement to zirconia. The highest bond strength was achieved using SIE combined with Panavia SA Plus cement. Increased surface roughness was observed with SIE, which contributed to the improved bond strength, which indicated that the choice of resin cement is crucial for achieving optimal results with both surface treatments [34].

Hot etching solution can increase fracture strength through various mechanisms. Treatment with hot etching for 60 min resulted in superior bond strengths compared to alternative methods. Utilizing a hot-etching solution for the surface treatment of zirconia may augment surface roughness and the bond strength between zirconia and resin cement. Moreover, evidence showed that hot etching was more suitable than APA for preparing the zirconia surface because it did not create microcracks in the specimens [35]. The differences in the findings can be attributed to variations in APA duration, hot etching duration, and cement type. However, even in studies using the same cement, different surface treatment methods resulted in varying outcomes [36,37].

The current study showed a higher mean of fracture strength in grooved surfaces treated on crown or abutment than hot etching or APA. The lowest retention was in the APA group (control), while this group showed higher fracture strength than specimens treated with hot etching. Studies reported various findings due to applying APA treatment for bonding, which has been related to using different particles, pressure, and time [38]. El-Korashy and El-Refai reported that APA yielded results slightly superior to hot etching by emphasizing silica coating for preparing the zirconia surface. It was noted that the high fracture strength following surface preparation with phase change silica coating could be attributed to the significant conversion from tetragonal to monoclinic phase. The phase transition, resulting in a 4 % volume increase, generated compressive stresses under external pressures such as grinding or APA, effectively inhibiting crack propagation or formation that contributed to the heightened fracture strength of zirconia compared to other ceramics [39]. Shimizu et al. reported that zirconia specimens prepared with APA showed superior bond strength compared to those prepared with low-pressure plasma [40].

A study by Russo et al. illustrated that among the pre-treatment methods, APA and tribo-chemical silica coating stand out with substantial evidence. The physicochemical conditioning of zirconia typically leads to enhanced bonding. Conversely, surface treatment has been shown to affect bonding outcomes detrimentally [7,40]. However, despite extensive research, there remains a lack of evidence supporting a universally applicable surface treatment protocol. The clinical implications of these findings are significant. The demonstrated effectiveness of grooving and hot etching suggests that these methods can be reliably used to enhance the retention of zirconia restorations, which is crucial for their long-term success. However, clinicians should be cautious with hot etching, as it might reduce fracture strength, especially in areas of high mechanical stress. One of the limitations of this study is the lack of surface characterization regarding roughness and contact angle. These parameters are crucial for understanding the effects of surface preparation methods on the retention and fracture strength of zirconia restorations. Future studies should consider these characteristics to provide a more comprehensive understanding of the results. Furthermore, the *in vitro* nature of this study might result in different outcomes compared to the clinical evaluations.

## 5 Conclusion

The study findings demonstrated that hot etching and grooving emerged as practical techniques for enhancing retention regardless of the substrate on which they were applied, with hot etching yielding the highest retention. While fracture strength remained relatively unaffected by surface preparation methods, it was notably enhanced using the grooving method. These findings underscored the importance of carefully selecting surface preparation methods to optimize the performance of Ti-base zirconia restorations.

## CRediT authorship contribution statement

**Mehran Falahchai:** Writing – review & editing, Writing – original draft, Supervision, Conceptualization. **Hamid Neshandar Asli:** Methodology, Investigation, Formal analysis, Data curation. **Mehdi Daneshvar:** Methodology, Investigation, Formal analysis, Data curation. **Amirreza Hendi:** Writing – review & editing, Writing – original draft, Supervision.

## Ethics approval and consent to participate

The study was approved by the ethical committee of the Guilan University of Medical Sciences, Rasht, Iran [IR.GUMS.REC.1399.660].

## Consent for publication

Not applicable.

## Availability of data and materials

The datasets are available from the corresponding author upon request.

## Declaration of generative AI in scientific writing

While preparing this work, the authors used ChatGPT to improve the manuscript's language and grammar. After using this tool/service, the authors reviewed and edited the content as needed and took full responsibility for the publication's content.

## Funding

No funding.

## Declaration of competing interest

The authors declare that they have no known competing financial interests or personal relationships that could have appeared to influence the work reported in this paper.
